# Supplementary figures and images for: Optimal cultivation measures for maize production in the drylands of the Loess Plateau
Source: PeerJ. 2025 Jul 16;13:e19654. doi: 10.7717/peerj.19654 (PMC12275898; doi:10.7717/peerj.19654)

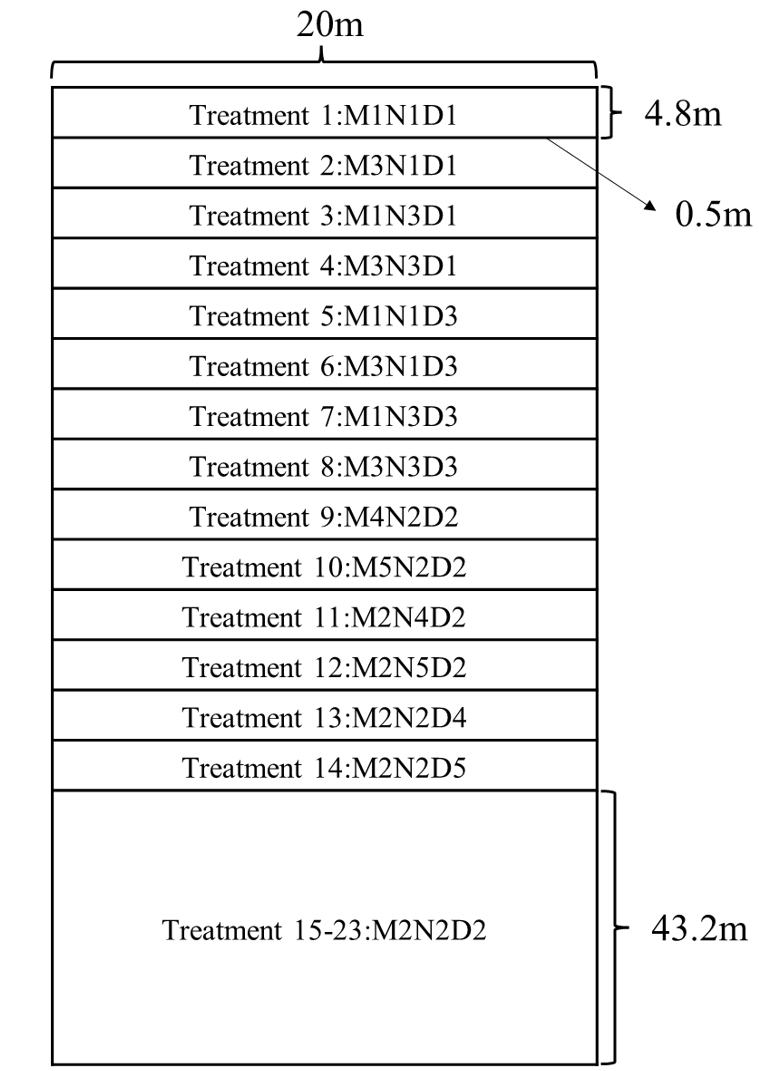

Supplement: Supplemental Information 1 [file peerj-13-19654-s001.zip › supplementary materials/supplementary figure 1.png]
